# Hysterosalpingographic evaluation of primary and secondary infertility

**DOI:** 10.12669/pjms.315.7545

**Published:** 2015

**Authors:** Muhammad Usman Aziz, Saleha Anwar, Syed Mahmood

**Affiliations:** 1 Muhammad Usman Aziz, MBBS, FCPS (Radiology). Senior Registrar, Department of Radiology, Liaquat National Hospital, National Stadium Road, Karachi, Pakistan; 2 Saleha Anwar, MBBS, FCPS (Radiology). Assistant Professor, Department of Radiology, Liaquat National Hospital, National Stadium Road, Karachi, Pakistan; 3 Syed Mahmood, MBBS, FCPS (Radiology). Karachi X-Rays and Diagnostic Center, M. A. Jinnah Road, Karachi, Pakistan

**Keywords:** Hysterosalpingography, infertility

## Abstract

**Objective:**

To determine the pathological patterns of fallopian tubes and uterus on hysterosalpingogrphy (HSG) examination in cases of infertility.

**Methods:**

Two years retrospective charts review of patients referred to our centre for HSG evaluation of infertility, from July 2008 to July 2010.

**Results:**

Four thousand one hundred eight hysterosalpingograms were carried out at our centre during the study period. Out of these, 1999 (48.6%) were primary infertility cases while the 2109 (51.3%) were of secondary infertility. Mean age of presentation for primary infertility was 30 years and 35 years for secondary infertility. Bilateral free peritoneal spill was noted in 60% of cases. Unilateral tubal blockage was present in 15% and bilateral tubal blockage in 10% of patients. Bilateral hydrosalpinx was present in 10% of patients and unilateral loculated spill was found in 5% of patients with primary infertility. Patients with uterine congenital anomalies were also evaluated and the frequency of bicornuate uterus was 4%, unicornuate uterus was 2% and uterine didelphys was 0.2%.

**Conclusions:**

Infertile patients who underwent HSG were mostly in older age group with secondary infertility being slightly more common emphasizing early work up and care. Most of the patients with primary infertility had normal HSG examination. To our knowledge this is the largest data for HSG to be presented from Pakistan.

## INTRODUCTION

According to The World Health Organization, infertility is defined as:^1^ “a disease of the reproductive system defined by the failure to achieve a clinical pregnancy after 12 months or more of regular unprotected sexual intercourse.”

Tens of millions of people worldwide suffer from infertility.^2^ Infertility rates have remained stable in the last few decades independent of the voluntary global decline in the number of preferred children with primary infertility showing a minimal decline of 0.1% and secondary infertility with a slight increase of 0.4%.^2^ Different factors are attributed to infertility including male factor, ovulation problems and uterine and tubal pathologies.^3^,^4^ Tubal causes are attributed to both primary and secondary infertility with higher prevalence in secondary type making routine tubal evaluation in secondary infertility a recommendation.^5^,^6^ Uterine causes of infertility include polyps or fibroids, uterine wall irregularities and congenital anomalies. Up to 50% of patients with recurrent failed pregnancies have shown uterine abnormalities in one study.^4^

Hysterosalpingography (HSG) also known as uterosalpingography investigates the morphology of uterine cavity and the patency of fallopian tubes and is a widely used radiological procedure. Evaluation for infertility and congenital uterine malformations are among the common reasons for ordering an HSG examination.^7^

This study evaluates the radiological pattern at HSG in female infertility patients in a two year period. To our knowledge this is the largest data for HSG to be presented from Pakistan.

## METHODS

This is a two year retrospective review of patients referred to the Karachi X-Rays and Diagnostic Center for HSG evaluation of infertility from July 2008 to July 2010. Review of only radiological records retrospectively meant no contact with the patients or their families during the course of this study. No contact was made with any other physician. As there was no ethical issue identified, ethical review board approval was not required. Data was entered into a standardized, pre tested data sheet.

Only undiagnosed patients with primary and secondary infertility were included in the study. Inconclusive examinations and studies not done for infertility were also excluded. Histories of recent intrauterine intervention and previous salpingectomy, ongoing vaginal discharge or bleeding and severe contrast allergy are the contraindications to HSG at our department. Our department protocol requires all HSG examinations to be performed after consent and in the first half of the menstrual cycle because of a thin endometrium which enables better image evaluation and also excludes early pregnancy.^8^ Also, approximately 10 – 15 ml of water soluble contrast material is injected following cannulation after all aseptic measures. Antibiotics may be prescribed to prevent infection. A control film of the pelvis is acquired for positioning and technical factors. With the patient supine, further films including anteroposterior and oblique views are taken. Cervix, uterine cavity, tubes and peritoneal spillage are visualized. Additional films may also be taken.

All the HSG examinations were analyzed and reviewed by two qualified radiologists and decisions were made by consensus. The available soft copy images were reviewed at the work station.

Normal uterine cavities with no structural abnormalities and filling defects and normal caliber fallopian tubes with free spillage of contrast into peritoneal cavity were declared normal examinations. Patients in whom the contrast failed to outline both or one tube and with no subsequent spillage were considered to have bilateral or unilateral tubal blockage while those with dilated tubes were classified as having hydrosalpinx. Absence of free flow and dispersion of contrast into the peritoneum defined loculated spill.

## RESULTS

A total of 4108 hysterosalpingograms were carried out at our centre for infertility during the study period. Out of these, 1999 (48.6%) were primary infertility cases while 2109 (51.3%) were of secondary infertility. Most of the patients with primary infertility had normal HSG examination. Mean age of presentation for primary infertility was 30 years and for secondary infertility was 35 years (Table-I). Bilateral free peritoneal spill was noted in 60% of cases.

**Table-I:** Classification and age distribution.

	Primary Infertility	Secondary Infertility
Number	1999	2109
Percentage	48.6	51.3
Mean age (years)	30	35

**Fig.1:**
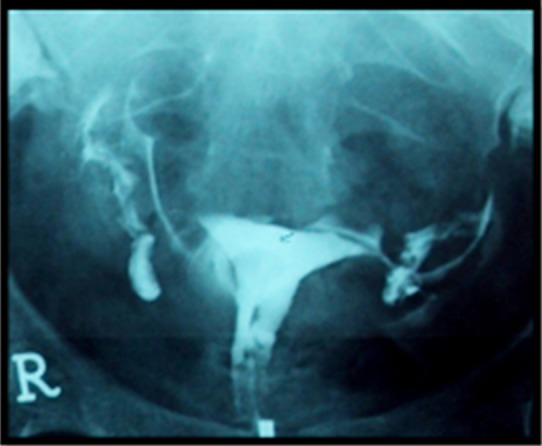
Normal HSG examination normal uterine cavity and bilateral fallopian tubes with normal free spillage of contrast on either side.

**Fig.2:**
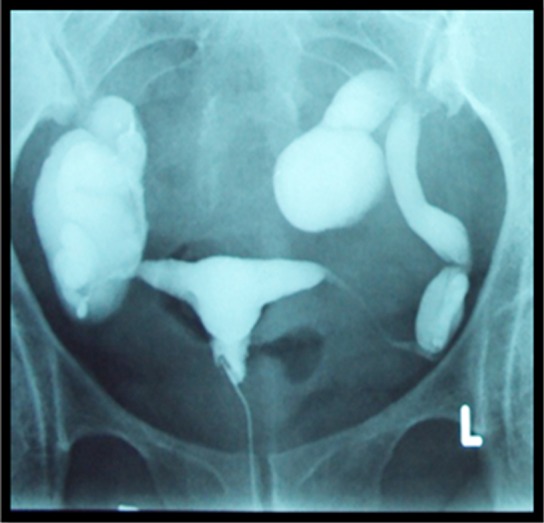
HSG showing bilateral tubal blockage resulting in hydrosalpinx.

**Fig.3:**
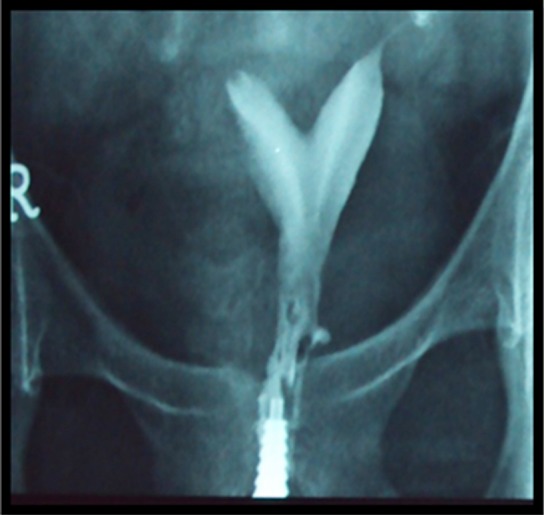
HSG of secondary infertility patient showing bicornuate uterus.

**Fig.4:**
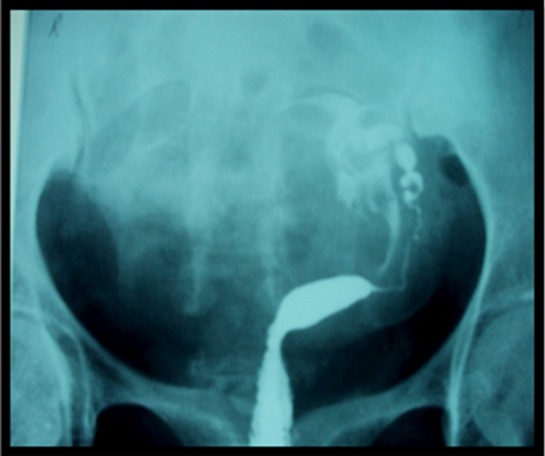
Unicornuate Uterus.

Unilateral tubal blockage was present in 15% and bilateral tubal blockage in 10% of patients. Bilateral hydrosalpinx was present in 10% of patients while unilateral loculated spill was found in 5% of patients with infertility. Patients with uterine congenital anomalies were also evaluated and it was found that the frequency of bicornuate uterus was 4%, unicornuate uterus was 2% and uterine didelphys was 0.2% in the present series (Fig. 1-4).

## DISCUSSION

Infertility is among the most widespread women’s health challenges in the developing countries. At the same time not much attention is paid to it.^9^ Poverty, pollution, lack of education, gender inequality and infectious diseases all contribute to high infertility rates.^9^ Population control has also played an adverse role.^10^ Children are associated with happiness, well being and social security.^11^ Childlessness often leads to adverse social and societal consequences.^9^,^12^,^13^

In agreement with earlier works,^6^,^14^ this study confirms secondary infertility being more common than primary (51.3% vs. 48.6%). Primary infertility has also been found to be more prevalent than secondary in other studies.^15^-^17^ Increased number of secondary infertility patients could be due to inadequate care during previous pregnancies or previous abortions resulting in pelvic infections.^6^,^14^ Tubal damage can be secondary to chlamydia infection and previous pelvic surgery among other factors.^18^,^19^ Chlamydia screening in the very first year of infertility has also been recommended pointing towards its high prevalence even in asymptomatic patients.^20^ Tubal abnormality (blockage, dilatation) was found to be the most common cause of infertility in this study. This is similar to other reports.^17^,^21^ Tubal pathologies have historically been the major cause of infertility.^22^ Differentiation of spasm from tubal blockage is also an important factor to be conscious of. Smooth margin versus irregularity is a useful pointer.^23^ Antispasmodics were used to prevent spasm in our study as recommended.^24^ Increased incidence of hydrosalpinx on right side has been observed in other studies due to the presence of appendix.^25^ We did not observe it in our study. Pelvic abnormalities like adhesions result in loculated spills and we found 5% (205) of patients with loculated spills.

Congenital anomalies were seen in 6.2% of cases (255). This is higher than reported by other authors.^26^,^27^ Commonest abnormality found was bicornuate uterus (4% - 164 cases). This is similar to few reports from Nigeria.^28^,^29^ The second commonest anomaly was unicornuate uterus (2% - 82% patients). Uterine synaechia was found to be the most common acquired uterine abnormality by other writers.^28^,^30^ again suggesting inadequate care during previous pregnancies.^28^ We did not study this.

Most of the patients with primary infertility had normal HSG examination suggesting the reason being other than physical. Male infertility workup is also often not done due to various reasons including infertility considered only as a female problem^31^ hence potentially contributing to a high number of normal HSG examinations in primary infertility. This has been observed earlier as well.^14^

Hysterosalpingography is an important part of infertility work up in our part of the world. It has been recommended as a screening tool in cases of infertility in developed countries as well.^32^ Other modalities like contrast sonohysterography, laparoscopy and hysteroscopy have been shown to be of comparable or more usefulness.^33^-^35^ HSG has also been found to be less sensitive than other modalities in cases of extratubal pathologies.^29^,^33^,^35^ However its wide availability compared to other modalities and a possible therapeutic role^36^ will help HSG remain the main diagnostic study in infertility cases.

Generalization of our study’s results is not recommended this being a single center study. No follow up was available for any case. Laparo- and/or hysteroscopic evaluations were also not available. This reflects upon the accuracy of HSG findings.

## CONCLUSION

HSG remains the diagnostic backbone for infertility although more frequent use of newer modalities should be encouraged. Infertile patients who underwent HSG were mostly in older age group with secondary infertility being slightly more common. These points towards the need for more emphasis on early evaluation and treatment. Proper practice and care should be ensured during pregnancies to prevent subsequent infections and possible tubal abnormalities. Routine chlamydia screening is another recommendation to be looked at. Also, male infertility workup is recommended through more education and awareness.
